# Intra-Articular Drug Delivery for Osteoarthritis Treatment

**DOI:** 10.3390/pharmaceutics13122166

**Published:** 2021-12-15

**Authors:** Yifeng Cao, Yifeng Ma, Yi Tao, Weifeng Lin, Ping Wang

**Affiliations:** 1 College of Pharmaceutical Science, Zhejiang University of Technology, Hangzhou 310014, China; yifengcao@zjut.edu.cn (Y.C.); 201706030612@zjut.edu.cn (Y.M.); taoyi1985@zjut.edu.cn (Y.T.); 2 Department of Molecular Chemistry and Materials Science, Weizmann Institute of Science, Rehovot 76100, Israel

**Keywords:** osteoarthritis (OA), developing disease-modifying OA drugs (DMOADs), intra-articular (IA) injection, drug delivery systems

## Abstract

Osteoarthritis (OA) is the most prevalent degenerative joint disease affecting millions of people worldwide. Currently, clinical nonsurgical treatments of OA are only limited to pain relief, anti-inflammation, and viscosupplementation. Developing disease-modifying OA drugs (DMOADs) is highly demanded for the efficient treatment of OA. As OA is a local disease, intra-articular (IA) injection directly delivers drugs to synovial joints, resulting in high-concentration drugs in the joint and reduced side effects, accompanied with traditional oral or topical administrations. However, the injected drugs are rapidly cleaved. By properly designing the drug delivery systems, prolonged retention time and targeting could be obtained. In this review, we summarize the drugs investigated for OA treatment and recent advances in the IA drug delivery systems, including micro- and nano-particles, liposomes, and hydrogels, hoping to provide some information for designing the IA injected formulations.

## 1. Introduction

**Figure 1:**
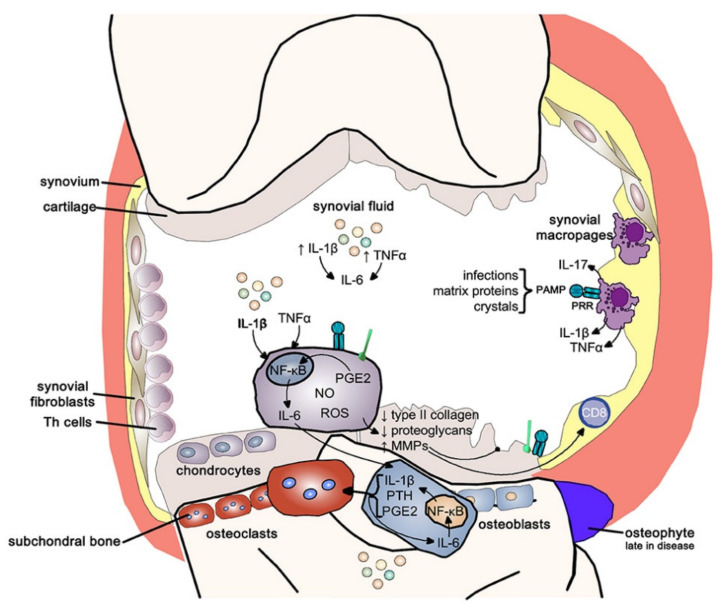
Structures of normal (left part of the figure) and osteoarthritic (right part) cartilages, as well as inflammation pathways in OA. Reprinted with permission from Bennett et al, Front. Med., published by Frontiers Media, 2018.

Healthy synovial joints are capable of maintaining extraordinary lubrication, load-bearing, and anti-wear functions for a life-long time, attributed to their characteristic structures (as schemed in Figure 1), as well as the cellular and molecular constitutions [1,2]. Osteoarthritis (OA), characterized by progressive damage of cartilage, and even the whole joint, is the most prevalent degenerative joint disease worldwide, particularly among elder people [3]. The onset of OA is the result of one or combined factors, including age, overweightness, joint injury, and infection [4,5,6]. Risk factors also include other diseases, such as type 2 diabetes [7]. Symptoms of OA include joint pain, swelling, mobility problems, and even disabilities, which not only affect patients’ daily activities and quality of life, but also have become a vital cause of disability, giving rise to great economic burden to patients and to socioeconomic cost [8].

Unfortunately, current clinical treatments for early OA are limited to symptomatic treatment to relieve the pain and inflammation, or viscosupplementation treatment to improve the viscoelastic property of synovial joints [9]. For late hip or knee OA, total joint replacement is the most feasible option, from which one third of the patients may still suffer from chronic pain and ca. 20% poor outcome [10,11]. It is highly demanded to develop efficient treatments for OA patients to prevent the cartilages from further damage or even repair the damaged cartilages. Herein, we review the clinically available and potential drugs for the treatment of OA, followed by summarizing recently developed drug delivery systems for IA injection.

## 2. Synovial Joints

A typical synovial joint consists of four parts: articular cartilage that covers the end of the bone, a synovial cavity filled with synovial fluid, a synovial capsule that seals the joint space, as well as a ligament connecting two articulating bones. Articular cartilage is an avascular tissue composed of sparsely distributed chondrocytes and a dense extracellular matrix (ECM), whereas ECM is a mixture of water (65–80%), collagen, proteoglycans, and glycoprotein, as well as hyaluronic acid (HA, also called hyaluronan), et al.; more importantly, it provides a super-lubricated surface and facilitates load transmission [12]. Once damaged, the articular cartilage can barely regeneratively repair by itself as there are no blood vessels in it. Synovial fluid is a viscous non-Newtonian fluid consisting of water, hyaluronic acid (greatly controlled the viscoelastic properties of synovial fluid), lubricin, proteoglycan 4 (PRG4), and phospholipid, which contribute to the boundary lubrication of the articular cartilage interface [13]. Fibroblast-like synoviocytes lining the synovium, the inner surface of synovial capsule, can secrete lubricants (proteoglycan, phospholipids, et al.), nutrient factors, and other substances [14].

A healthy synovial joint can provide extremely low friction coefficients (μ = frictional force/normal load) 0.01−0.003 under physiologically high pressures up to 5 MPa, or even higher [2,15,16]. It is generally accepted that a mixed lubrication mechanism, fluid-film lubrication and boundary lubrication, accounts for such extraordinary lubrication [17]. In the case of fluid-film lubrication, a viscous thin film separates the articulating surfaces; therefore, it is crucial to maintain the characteristic viscoelastic properties of synovial fluid. As for boundary lubrication, molecules in the boundary layer are in close contact and determine the lubrication behavior; phospholipids (PLs), which line the cartilage surface and are complexed with underlying HA and lubricin molecules, expose their highly hydrated headgroup layer to the interface and reduce friction coefficients by a hydration lubrication mechanism when two such surfaces slide past each other [1,18,19,20,21,22].

## 3. Medication Therapies for OA

### 3.1. Drugs for the Treatment of OA

Chronic pain is associated with OA disease. Symptomatic treatment to relieve the pain has been the prime option in clinic. For this purpose, IA injection of corticosteroid, including glucocorticoid, has been a favorable treatment in the clinical treatment of OA and is recommended by the guidelines updated in 2019 by the European Society for Clinical and Economic Aspects of Osteoporosis, Osteoarthritis and Musculoskeletal Diseases (ESCEO) and by the Osteoarthritis Research Society International (OARSI) [9]. IA injection of corticosteroid shows moderate pain relief and limited function improvement, accompanied by low-rate complications, such as accelerated OA progression [23,24,25].

When OA occurs, the molecular weight of HA in synovial fluid decreases, resulting in less viscous synovial fluid, which is not favorable for the lubrication behavior. Adding HA as a viscosupplement can restore the viscoelastic characteristics of synovial fluid [26]. Approved by the Food and Drug Administration (FDA), IA injected HA molecules or cross-linked HA has been clinically administered to OA patients [9,27]. However, from the lubrication point of view, HA molecules are not good boundary lubricants [28], but they are essential in facilitating the adsorption of PLs by forming complexes, which synergistically reduces the friction coefficients between two sliding surfaces [18,29,30,31]. 

In addition to gradually damaged cartilages, OA is also characterized by a narrowed synovial cavity, invasion in the subchondral bone, and formation of osteophyte, as well as inflammation in the synovial membrane and synovial fluid [32]. However, the pathogenesis of OA is complicated and has not been fully revealed until now. OA has long been considered to be a “wear-and-tear” disease, wherein lesions of the articular cartilage have been considered to be the main inducements of OA [33]. In the past decades, the onset and progress of OA have generally been recognized as inflammation-involved processes taking place not only in chondrocytes, but also in other tissues of the joints [34,35]. Additionally, the innate immune system is also involved in the pathogenesis of OA [36,37]. Under mechanical stimulation or when exposed to inflammatory cytokines, chondrocytes in the cartilage, fibroblast-like synoviocytes, as well as osteoblasts in the subchondral bone, are all able to express inflammatory cytokines, including interleukin (IL)-1β, IL-6, fibroblast growth factor 2 (FGF2), matrix metalloproteinase 3 (MMP-3), and tumor necrosis factor α (TNFα), as well as matrix-degrading enzymes, such as collagenases and aggrecan-degrading enzymes, which contribute to cartilage degradation (Figure 1) [32,38,39]. Proinflammatory cytokines, including IL-1β and TNF, also provide substantial targets for the development of disease-modifying drugs [40]. Evidence indicates that some biological agents can act on their intended primary targets and potently induce immune tolerance [41]. For the treatment of rheumatoid arthritis (RA), an autoimmune disease that primarily causes joint inflammation, induction of immune tolerance, possibly on cytokines, is one of the emerging therapeutic strategies [42,43]. OA treatment might also benefit from this strategy, probably to a less significant extent than in the case of RA.

As inflammation is indicated in the pathogenesis of OA, topical and oral administration of nonsteroidal anti-inflammatory drugs (NSAIDs), including but not limited to ibuprofen, salicylate, and diclofenac, have been used for the anti-inflammatory purpose, which also relieve pain and stiffness in the joints [9,44]. HA can not only reduce friction coefficients/mechanical friction damage on the surface of the joint, as mentioned before, but also protect the synovial tissue from the influence of inflammatory mediators [45,46].

According to the pathogenesis of OA, disease-modifying OA drugs (DMOADs) have been developed in the following approaches [47,48]: (a) protecting chondrocytes from catabolic activity with antagonists of IL-1b and TNF-a (for example, IL-1 receptor antagonist (IL-1 Ra), a natural IL-1 inhibitor binding to IL-1 receptor [49]), and from inhibited autophagy by inhibitors of mammalian target of rapamycin (mTOR, the main negative regulator of autophagy) [50] and by microRNAs (miRNAs) [51]; (b) slowing down or preventing the destruction of cartilage by inhibiting matrix-degrading enzymes, one such drug being the anti-ADAMTS (short for a disintegrin and metalloproteinase with thrombospondin motifs)-5 Nanobody^®^ M6495 [52]; (c) facilitating the regeneration of articular cartilage by anabolic agents, such as FGF18 (for example, sprifermin, recombinant human FGF18 [53]), which promote chondrocyte and chondrocyte progenitor cell proliferation [54]; (d) based on small-molecule effectors of endogenous stem cell populations, such as kartogenin (KGN) [55]. As far as we know, none of the DMOADs have been approved by the FDA, but some of them are under clinical trials. In addition to the above-mentioned drugs for the treatment of OA, including symptomatic treatment for pain relief and anti-inflammation, viscosuppliment, and DMOADs, platelet-rich plasma (PRP) treatment and stem cell injections both show great potential in OA treatment [56,57]. However, they are strongly recommended against by the American College of Rheumatology and the Arthritis Foundation (ACR/AF) and OARSI, mainly due to heterogeneity and lack of standardization [58,59].

### 3.2. Routes of Drug Administration

The route of drug administration largely determines the effectiveness and safety, and, in particular, the therapeutic efficacy of drugs [60]. Oral and topical/transdermal administrations are the most widely used routes in the treatment of early-stage OA. By oral administration, the active ingredients, such as NSAIDs, reach joints through the circulatory system. Only a small amount of drug can reach the designated site due to the cartilage being devoid of blood vessels. In addition, as OA is a local but not systematic illness, oral administration is also facing problems such as low bioavailability, gastrointestinal injury (for example, bleeding and ulceration), as well as perforation of the stomach, small intestine, or large intestine, and even liver and kidney damage [61,62]. Topical/transdermal administration in the form of emulsion or patches can remarkably reduce toxicity and irritation, but less drug is able to cross skin barriers [63]. Additionally, although topical administration of NSAIDs shows superior pain-relief efficiency over placebo, its efficacy is limited to two weeks [44]. To overcome the problem with low skin permeability, microneedle, a novel transdermal drug delivery device that can pierce the dermis and deliver drugs into the systemic circulation without passing skin barriers, can be used [64]. 

Soluble small and large molecules in the circulation system enter the joint cavity through the synovial capillaries, resulting in low bioavailability. IA injection, which directly delivers drugs to the joint, shows some advantages, including improved utilization and enhanced efficacy of drugs, faster relieved symptoms, and reduced systemic adverse reactions. As a result, IA injection of drugs for the treatment of OA has attracted more and more attention. Both small and large molecules are rapidly cleaved in the joint by small blood vesicles and the lymphatic system, respectively [27]. Therefore, a proper drug delivery system for IA injection is highly demanded.

## 4. Intra-Articular (IA) Drug Delivery Systems

The effectiveness of drugs for the treatment of OA is determined not only by the administration route, but also by the drug formulation [60]. As IA injection of drugs is limited by the poor permeability of drugs into the cartilage and the rapid clearance of drugs, particularly small-molecular ones, from the joint through synovial capillaries and lymphatics [27], various drug delivery systems for IA injection, such as nanoparticles, liposomes, and gels, have been developed to prolong the bioavailability and circulation time to improve the efficacy of drugs (Table 1). Special consideration should be paid to the macrophage response to the injected materials, which leads to lowered drug bioavailability. Additionally, the carriers can be modified for targeting specific sites in the synovial joints, including cartilage, chondrocytes, ECM of cartilage, as well as synovium. For example, cartilage-targeted drug delivery can be obtained by electrostatic interactions between positively charged, drug-loaded vehicles and the negatively charged cartilage surface [65]. Valid targeting gives rise to rapid penetration, enhanced uptake, and retention time of drugs, and, therefore, enhanced therapeutic efficiency [66].

### 4.1. Microparticles (MPs)

Microcapsules and microspheres are the main forms of microparticles. Microcapsules are formed by encapsulating core material with polymeric capsules, while microspheres are microscopic spherical entities formed when drugs are dispersed or adsorbed in the polymer matrix. Microparticles as drug carriers show some favorable advantages, including slow release, long-term effect, and improved stability of drugs, but it is still limited by relatively low drug loading, complex production technology, and so on.

Microspheres prepared from polymers undergoing emulsification-solvent evaporation are a feasible way of preparing MPs. Biocompatible synthetic polymers, including poly(lactic-co-glycolic acid) (PLGA) [67], poly ethyl amide (PEA) based on α-amino acids, aliphatic dicarboxylic acids, and aliphatic α-ω diols [73], as well as poly(3-hydroxybutyrate-co-ε-caprolactone) copolymers (PHBCL copolymers) [76], have been reported as matrixes for IA injected drugs. Zhang et al. [67] have prepared microspheres encapsulating tetramethylpyrazine (TMP) with PLGA using an emulsion/solvent evaporation method. Results confirmed that IA injection of the microspheres can significantly prolong the residence time of TMP in the articular cavity. Park et al. [71] also confirmed the positive effects of fabricated ibuprofen-loaded PLGA porous microspheres (IBU/PMSS) in inhibiting inflammation and alleviating pain. Compared with ordinary microspheres, porous microspheres have many advantages, such as more pores, light texture, strong bearing capacity, and fast diffusion rate. Meanwhile, sustained release of ibuprofen for up to 63 days was observed. An autoregulatory drug delivery system prepared from celecoxib-loaded PEA microspheres was reported by Janssen et al. [73], whereas the encapsulation of celecoxib significantly inhibited the degradation of PEA microspheres. After a short burst release, slow release of celecoxib for >80 days was observed. Rudnik-Jansen et al. [74] compared triamcinolone acetonide (TAA)-loaded PLGA microspheres and PEA microspheres. Although both of them can significantly reduce synovitis and alleviate pain, carriers prepared from PEA were more effective and durable than the PLGA-based ones. Besides, Musumeci et al. [76] prepared a microparticle delivery system containing water-soluble diclofenac sodium for the treatment of OA using hydrophobic biodegradable PHBCL copolymers by the solvent deposition method. The ratio of HB/CL, as well as molecular weight, influence the structure, dimension, and shape of the formed MPs. An initial burst release, followed by a slow release for up to 72 h, indicated a diffusion-controlled drug release pattern. Additionally, this copolymer was considered neither toxic nor genotoxic under the desired concentration.

Natural macromolecules, such as gelatin [78], silk fibroin [78,116], and heparin [81], being biocompatible and biodegradable, have also been investigated as building blocks for intra-articularly injected microspheres. Ratanavaraporn et al. [78] developed gelatin or gelatin/silk fibroin microspheres loaded with curcumin. Results show that, compared with rapid-degrading gelatin microspheres, gelatin/silk fibroin microspheres degraded slowly and, therefore, showed a longer anti-inflammatory effect by reducing the level of IL-6. In addition, the clonoxicam-loaded chitosan microspheres crosslinked with anionic tripolyphosphate (TPP) prepared by Abd-Allah et al. [79] also showed long-term anti-inflammatory effects in vivo. Interestingly, Tellier et al. [81] prepared a microparticle system based on heparin for the delivery of Hep-N tumor necrosis factor-alpha stimulated gene-6 (TSG-6). In addition to reduced cartilage injury and protection of the bone and joint, heparin sulfation positively enhanced the bioactivity of TSG-6.

**Figure 2:**
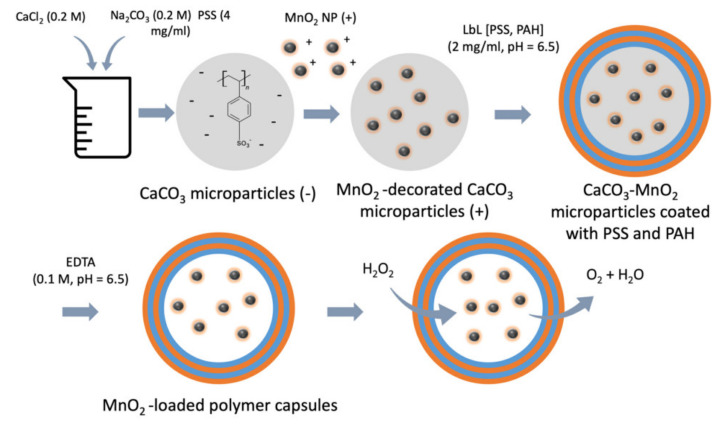
Synthesis and effective diagram of antioxidant microcapsules loaded with manganese dioxide. Reprinted with permission from Marin et al, Mater. Sci. Eng. C Mater. Biol. Appl, published by Elsevier, 2020.

In addition to microspheres, microcapsules are receiving increasing attention in the treatment of OA. Lengert et al. [83] have developed a new formulation of betamethasone-dipropionate-loaded hollow silver hydrogel microcapsules by an aqueous-based approach. In vitro experiments showed that the micro-spherical delivery system can improve the bioavailability and effectiveness of betamethasone dipropionate when compared with free drug. However, silver nanoparticles (AgNPs) on the shell caused concentration-dependent cytotoxicity to L929 cells. The accumulation of reactive oxygen and nitrogen species (ROS and RNS) in the cell microenvironment can cause oxidative stress, leading to cell apoptosis and senescence, which negatively affects OA [117]. Thus, an antioxidant approach is another strategy for alleviating OA. Marin et al. [84] used manganese dioxide nanoparticles (MnO_2_ NPs) as a more effective inorganic substitute for antioxidant enzymes and fabricated an antioxidant microreactor by layer-by-layer (LbL) polymer microcapsules encapsulating MnO_2_ NPs (Figure 2). The LbL shell controls the diffusion of chemicals. These microreactors efficiently protected cells from oxidative stress by scavenging hydrogen peroxide (H_2_O_2_) from solution.

### 4.2. Nanoparticles (NPs)

The performance of IA injected drug carriers is size-dependent [118,119]. Compared with microparticles, NPs have been used as carriers for various drugs/therapeutic agents and prolong the residence time of drugs in the target site. More significantly, NPs can penetrate biological barriers and improve the bioavailability of drugs [118,120,121,122,123,124,125]. Polymeric NPs, inorganic NPs (including metal NPs, silicon NPs, etc.), as well as proteins have been investigated as IA drug delivery carriers or active agents. Particularly, positively charged carriers, such as chitosan [119] and avidin [126], can take advantage of the electrostatic interaction between cationic carriers and negatively charged molecules in ECM of cartilage, enabling targeted delivery, rapid penetration, as well as enhanced uptake and retention of IA injected NPs [66].

Polymeric NPs are mostly prepared from emulsion polymerization, self-assembled amphiphilic block copolymers, as well as dendrimer synthesis methods [127]. IL-1 receptor antagonist (IL-1Ra) is a naturally occurring IL-1 inhibitor, which can bind to the IL-1 receptor and prevent it from playing a role in the inflammatory response [87]. Agarwal et al. [86] synthesized self-assembled poly(2-hydroxyethyl methacrylate)-pyridine nanoparticles presenting IL-1Ra on the surface. The nanocarriers had good biocompatibility and stability, thus improving the efficiency of drug utilization. Geiger et al. [90] reported amine terminal polyamidoamine (PAMAM) dendrimers end-functionalized with poly(ethylene glycol) (PEG) to control surface charge and conjugated to insulin-like growth factor 1 (IGF-1). PEG is the most widely used antifouling polymer, which can resist the nonspecific protein adsorption and protect the modified particles from aggregation and opsonization, therefore prolonging the circulation time of the particles. This cationic dendrimer nanocarrier exhibited improved targeting to chondrocytes, and prolonged the IGF-1 residence time for up to 30 days, thus improving its efficacy and slowing down cartilage degeneration.

**Figure 3:**
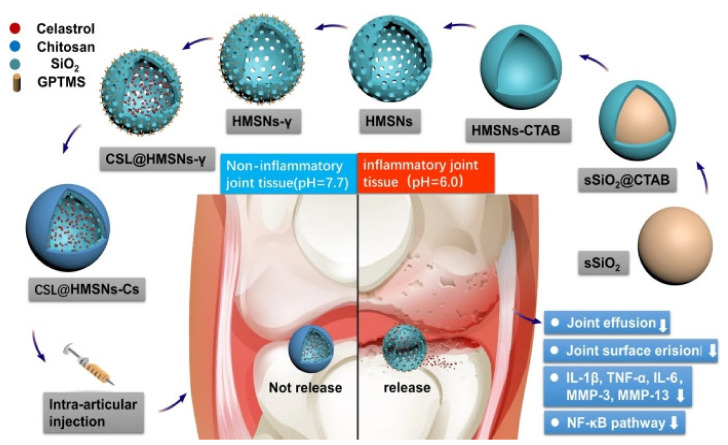
The process during which celastrol-loaded hollow mesoporous silica nanoparticles capping with chitosan (CSL@HMSNs-Cs) are synthesized, injected, released at a specific pH, and their effectiveness. Reprinted with permission from Jin et al, J. Nanobiotechnol., published by Springer Nature.

Hollow mesoporous silica nanoparticles are promising drug delivery nanocarriers, with their huge loading capacity and good biocompatibility. Jin et al. [91] prepared hollow mesoporous silica nanoparticles (HMSN) by loading celastrol (CSL), a natural bioactive compound that can protect chondrocytes against IL-1β-induced inflammation response and apoptosis [128], and capping with chitosan (Cs) to respond to the acidic environment of inflammatory joints (Figure 3). The low toxicity of nanoparticles and the improvement of articular surface erosion and joint effusion were confirmed. Another group of functionalized mesoporous silica-based drug carriers for treating OA was developed by Zhang et al. [93,94]. They constructed nanospheres by loading diclofenac sodium (DS) into super-lubricated poly(2-methacryloyloxyethyl phosphorylcholine)-grafted mesoporous silica nanoparticles (MSNs-NH_2_@PMPC-DS). The remarkable advantages of the nanospheres include a high drug load and slow-release effect by MSNs, together with a super-lubricating effect on the joints, attributed to hydration lubrication, by PMPC moieties [19], which enable DS to exert its maximum potency in the joint [93]. Later on, they constructed supermolecules from azobenzene-modified mesoporous silica nanoparticles (bMSNs-AZO) and β-cyclodextrin-modified poly(2-methacryloyloxyethyl phosphorylcholine) (CD-PMPC), exhibiting light-responsive drug release and lubrication enhancement dual functions.

Gold (Au) compounds are also effective inflammatory cytokine inhibitors for the treatment of inflammatory diseases. The anti-inflammatory mechanism of gold in OA treatment includes the suppression of lysosomal enzymes released by phagocytic cells, modulation of prostaglandins, suppression of synovial cell proliferation, and promoting collagen synthesis [96,97,98]. Abdel-Aziz et al. [95] synthesized gold nanoparticles (AuNPs) and observed a synergistic therapeutic effect in combination with diacerein. Although AuNPs have been widely used in biomedical applications in recent years, the toxicity of gold nanoparticles has not been fully studied. AuNPs may exert cytotoxicity by inducing oxidative stress and damage to organs, such as liver, kidney, spleen, and lung, in vitro [129]. The toxicity of AuNPs depends on a number of parameters, including the configuration (shape and size), the concentration of AuNPs, as well as surface properties regulated by surface modification and biological corona [130,131]. Therefore, special attention should be paid to the utilization of AuNPs.

In addition, more and more NP drug carriers, such as nanocrystal–polymer particles (NPPs) [99] and metal–organic-frameworks (MOFs) [101], have been developed for IA treatment of OA. Maudens et al. [99] prepared NPPs by encapsulating high-payload KGN nanocrystals into poly(D,L-lactic acid)-Cyanine 7 (PLA-Cy7) particles. The experimental results showed that KGN-NPPs had a certain cartilage protective effect and a high drug loading, indicating that KGN-NPP was a promising delivery system. MOF is a newly developed class of porous material that has abundant metal sites to improve drug loading capacity [132,133,134] and has been utilized in pharmaceutics, biology, and other fields. Xiong et al. [101] have developed a pH-responsive MOF, MIL-100(Fe), loading with HA and protocatechuic acid (PCA). MIL-100(Fe) exhibits a high loading property, good biocompatibility, and pH responsiveness [135,136,137]. HA has also been proved to enhance the stability and dispersibility of MOF NPs. The weakly acidic environment of the inflamed joint is a critical stimulus triggering the precise release of drugs.

Nanodrugs improve their efficacy mainly through controlled-release formulations, increased plasma half-life, and bioavailability. Although it seems that they have a great application prospect, FDA-approved anti-inflammatory analgesic drugs are very scarce. The main reasons include, but are not limited to, complexity in the design of biocompatible nanomedicines, the potential toxicity of nanomaterials, and the difficulty of scaling up in industrial production [138]. Therefore, in order to develop safe and effective nanodrugs, future research should focus on solving these problems.

### 4.3. Liposomes

**Figure 4:**
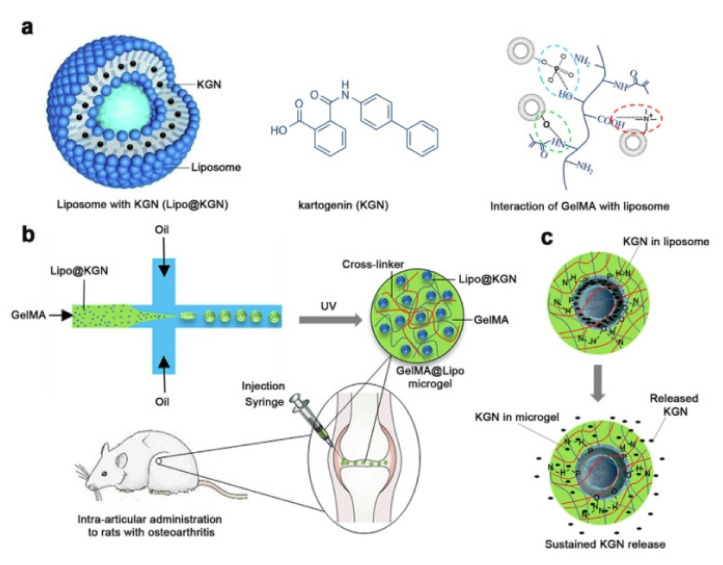
(**a**) Liposome loaded with kartogenin (KGN), chemical structure of KGN, and hydrogen bonds between GelMA and liposomes (from left to right); (**b**) preparation process and intra-articular injection of gelatin methacryloyl microgels incorporating KGN-loaded liposomes (GelMA@Lipo@KGN) to mice with OA; (**c**) sustained KGN release by overcoming the dual obstacles of lipid membrane and hydrogel matrix network. Reprinted with permission from Yang et al, Chem. Eng. J, published by Elsevier, 2020.

Similar to the cell membrane, a liposome is a closed spherical lipid bilayer typically prepared from phospholipids and cholesterol, which have also been identified in synovial joints [139,140]. Because of the amphoteric properties of phospholipids, liposomes (Figure 4a) are capable of encapsulating hydrophilic drugs (such as dexamethasone phosphate [141], a corticosteroid) in the interior aqueous space and/or hydrophobic drugs (such as KNG [114]) inserted in the hydrophobic region of the bilayer. In addition to tuning the physicochemical properties of liposomes by choosing different PL species and ratios, the outer surface also enables functional modifications to achieve long-term stabilized vesicles [142], targeted drug-delivery carriers [143,144], and theranostic platforms [145]. Together with their high biocompatibility, biodegradability, low toxicity, and cytotoxicity, as well as good lubricity, liposomes are fascinating and feasible drug carriers for IA drug delivery [144]. Liposomes are commonly prepared by methods such as sonication, repeated freeze–thaw, hydration–extrusion, and using microfluidic devices. Drugs are encapsulated in liposomes by passive loading, whereas drugs are firstly mixed with lipids, followed by the formation of vesicles, or by remote loading, which takes advantage of the transmembrane ion gradient [114,146]. In general, the fabrication process is easier for the former method, but higher drug-loading capacity and efficiency are achieved with the latter one.

NSAIDs, such as diclofenac, ibuprofen, indomethacin, and tenoxicam, are commonly used as anti-inflammatory treatments for OA patients. Pawar et al. [70] used thin-film hydration technology to prepare drug-fortified cationic liposomes of 6-methoxy-2-naphthylacetic acid (6-MNA). The prepared liposomes presented good biocompatibility in the OA site of rats, as well as sustained release of 6-MNA for >12 h. As the mammalian target of rapamycin (mTOR) inhibitor is a potential target for OA treatment, rapamycin can increase autophagy of human chondrocytes and prevent changes in OA-like gene expression in vitro [50]. Chen et al. [102] prepared liposome-encapsulated rapamycin (L-rapa) with a mixture of 1,2-distearoyl-sn-glycero-3-phosphorylcholine (DSPC), cholesterol, and positively charged octadecylamine by the thin-film hydration method. The encapsulation efficiency of rapamycin was 77.94 ± 8.5%, and sustained release of rapamycin within 72 h was obtained by L-rapa. Moreover, IA injection of L-rapa combined with low-intensity pulsed ultrasound (LIPUS) significantly enhanced the production of proteoglycan and type II collagen and reduced the MMP-13 levels, effectively reducing the dosage and frequency of administered rapamycin, as well as the adverse effects. Liposome size is an important parameter regulating the retention time: an increase in diameter from 160 to 750 nm improved the retention of dexamethasone palmitate (DMP) by a factor of 2.6 [147].

As mentioned earlier, highly hydrated phospholipids lining the surface of articular cartilage and acting as efficient boundary lubricants play a key role in the extraordinary lubrication behavior of synovial joints [1,18]. Besides delivering therapeutic agents to joints, IA injection of liposomal formulation also provides supplementary boundary lubricants, which could improve the lubrication behavior of the joints and substantially contribute to OA treatment. Liposomes stabilized against aggregation by incorporating lipid/poly(2-(methacryloyloxy)ethyl phosphorylcholine) (PMPC) conjugates provide efficient lubrication, superior to that of the most commonly used PEGylated liposomes [142]. Taken into the lubrication ability of drug carriers, liposomes hold potential for IA administration.

### 4.4. Hydrogels

Hydrogels provide three-dimensional and porous frameworks for the encapsulation and delivery of drugs, proteins, and cells [46]. Sinomenium is a kind of natural alkaloid with anti-rheumatism and immunomodulatory function [148]. Gelatin methacrylate (GelMA) is a kind of photopolymerizable hydrogel with the characteristics of cell adhesion domains [149], thermosensitivity [150], and enzymatic degradability [151]. Chen et al. [105] developed a photo-crosslinked GelMA hydrogel and combined it with sinomenium encapsulated by chitosan microspheres, which confirmed that this system has a good prospect in the treatment of osteoarthritis. The prepared drug delivery system showed controlled release of sinomenium and can improve cartilage degeneration by downregulating autophagy of chondrocytes. In addition to pain relief and anti-inflammatory drugs and DMOADs, cell therapy using mesenchymal stem cells (MSCs) is being explored as a potential treatment for OA. Xing et al. [110] developed a three-dimensional (3D) gelatin-based microcryogel loaded with low-dose human umbilical-cord-derived MSCs (hUC-MSCs), which was afterward proved to have better cartilage protection and repair effects according to trials on rats. Using microgels as carriers can enhance the survival and retention of low-dose cell and reduce side effects in osteoarthritic joints, while maintaining the similar therapeutic effects to those of high-dose free MSCs. Thermosensitive HA gels for IA injection of an anti-inflammatory agent, diclofenac potassium, were fabricated by Amira Hanafy and El-Ganainy [111]. They chose Poloxamer 407 as the main temperature-sensitive substance. In addition to cartilage regeneration, the thermosensitive gel formed after IA injection significantly extended the release period of the drug and retention time of HA. The good biodegradability and biocompatibility, easy chemical modification, relatively safe, and mature preparation technology of HA presented advantages for the wide application of HA in IA drug delivery systems [152,153,154,155].

Drug-loaded micro- or nano-particles, or liposomes embedded in hydrogels or microgels, taking advantage of the high drug load and long-term sustained release of drug-loaded particles/liposomes and good biocompatibility of hydrogel, are a group of attractive IA drug delivery systems. Stefani et al. [112] prepared an implant acellular agarose hydrogel embedded with dexamethasone (DEX)-loaded PLGA microspheres (DLMS), showing sustained DEX release for at least 99 days, as well as chondroprotection and improved joint functions. Another experiment using nanoparticles in combination with gels was conducted by Castro et al. [113]. Model small-molecule-loaded PLGA NPs were embedded in photo-crosslinked nanocomposite 4-arm-poly(ethylene glycol)-maleimide (PEG-4MAL) microgels, which show a prolonged residence time (>3 weeks) in the joint. They also proved that solid lipid NPs and gold NPs could be effectively loaded into PEG-4MAL microgels, providing a theoretical basis for the development of a new combination drug delivery system. Yang et al. [114] integrated KGN-loaded lectin–cholesterol liposomes into photo-crosslinked gelatin methacryloyl (GelMA) microgels (GelMA@Lipo@KGN) by microfluidics (Figure 4). Due to the obstruction of physical network and noncovalent interaction, liposomes were anchored in the microgel, which laid the foundation for its strong slow-release function. Experiments with rats showed that the hybrid microgel could significantly prolong the release time of KGN when compared with ordinary KGN-carrying liposomes. Moreover, it can effectively promote chondrocyte differentiation of bone marrow mesenchymal stem cells (BMSCs). Immobilizing nanoparticles and liposomes into the hydrogel matrix network has been an effective strategy to protect them from rapid clearance in vivo, which has promoted slow and persistent local delivery. Diclofenac-sodium-loaded alginate microspheres dispersed into injectable thermosensitive hydrogels made of chitosan and β-glycerophosphate were designed by Qi et al. [115]. Pharmacodynamic results showed that the combined administration system showed sustained drug release for up to 5 days, and could reduce the swelling of arthritic joints and relieve the symptoms of OA.

## 5. Conclusions

By rational design of drug delivery systems, IA injection of drugs shows great potential for efficient treatment of OA. Micro- and nano-particles, liposomes, and hydrogels are among the most widely investigated IA drug delivery systems. They play an essential role in improving the efficacy of active substances, mainly by controlled release and extended residence time, as well as targeting specific sites. Learning from the failures of DMOADs in clinical trials, we bring forward the following suggestions for future investigations: (1) understanding the pathogenesis of OA can provide more effective target sites for developing DMOADs; (2) these new drug delivery systems should also be evaluated for long-term chronic toxicity to ensure their safety over the long term; (3) combination therapy targeting more than one site/symptom may be a more efficient IA drug delivery strategy for OA therapy; (4) liposomes with drug delivery, lubrication, and targeting functions are promising drug-delivery carriers for intra-articular injection; (5) macrophage response to polymers, NPs, and MPs will lead to low drug bioavailability. Suitable antifouling coatings (coverage, surface charge, and surface hydrophilicity) should be incorporated to these polymers, NPs, and MPs to reduce the macrophage uptake of these materials.

## Figures and Tables

**Table 1:** Summary of information on different drug delivery systems investigated over the past five years.

Carrier/Materials	Size	Drug/Active Compound	Therapeutic Mechanism of Drug	Advantages of the Formulation	Ref.
Microparticles					
Poly(lactic-co-glycolic acid) (PLGA)	About 10 µm	Tetramethylpyrazine (TMP)	Alleviating IL-1-induced destruction of cartilage and chondrocytes, reducing the degradation of glycosaminoglycan, increasing chondrocyte activity, and inhibiting chondrocyte apoptosis; promoting chondrocyte proliferation by promoting cell cycle progression [67,68,69]	Prolong the retention time of TMP to 10 days	[67]
PLGA	ca. 250 µm	Ibuprofen	Inhibitor of cycloxygenase-2 enzyme [70,71]	Sustained drug release for 63 days, porous microspheres with light texture, strong bearing capacity, and fast diffusion rate	[71]
Polyester amide (PEA)	5, 15–20 µm (PDI 1.6)	Celecoxib	An anti-inflammatory drug that has been shown to be an effective analgesic for OA-related pain [72]	Auto-regulatory behavior, slow release for > 80 days, good intra-articular biocompatibility	[73]
PLGA, PEA	39.4 µm, PDI 1.30 (PLGA MPs) 23.8 µm, PDI 1.30 (PEA MPs)	Triamcinolone acetonide (TAA)	Inhibiting pain and inflammation for prolonged periods in OA knee joints [74,75]	Reduced synovitis and alleviating pain for at least 42 days	[74]
Poly(3-hydroxybutyrate-co-ε-caprolactone) (PHBCL) copolymers	0.5 and 4.5 µm	Diclofenac sodium	An anti-inflammatory drug that inhibits various enzymes and blocks the synthesis of prostaglandin [76,77]	Slow release	[76]
Gelatin, gelatin/silk fibroin	100–300 μm	Curcumin	Reduced the level of IL-6 in serum, delayed the cellular destruction in the articular joint and synovial tissue [78]	Lasting anti-inflammatory effect	[78]
Chitosan, tripolyphosphate (TPP)	3.57 to 6.12 µm	Lornoxicam	Inhibiting cyclo-oxygenase (COX), key enzyme of arachidonic acid pathway, thus inhibiting prostaglandin synthesis [79,80]	Long-term anti-inflammatory effects	[79]
Heparin	80 ± 60 µm	Hep-N tumor necrosis factor-alpha stimulated gene-6 (TSG-6)	Suppressing the response of chondrocytes to inflammatory factors, such as IL-1 and TNF-α [81,82]	Heparin sulfation significantly enhanced anti-plasmin activity of TSG-6, reducing cartilage injury, and protecting the bone and joint	[81]
Silver alginate microcapsules	1.3 ± 0.2 µm	Betamethasone dipropionate (Bm)	Inhibiting inflammation [83]	Improve the bioavailability and effectiveness compared to free Bm	[83]
Poly(sodium-p-styrene sulfonate)/poly(allylamine hydrochloride) (PSS-PAH)	ca. 50 nm	MnO_2_	MnO_2_ nanoparticles can be used as reactive oxygen scavengers that mimic catalase and superoxide dismutase (SOD) activity simultaneously [84,85]	Robust capsules capable of multiple re-use and resisting ethanol sterilization	[84]
Nanoparticles					
Poly(2-hydroxyethyl methacrylate)-pyridine	300–700 nm	IL-1Ra	Binding to the IL-1 receptor (IL-1R) without triggering an agonist response, and thus functioning as a receptor antagonist [86,87]	Good biocompatibility and stability	[86]
PEGylated cationic polyamidoamine (PAMAM) dendrimer	<15 nm	Insulin-like growth factor 1 (IGF-1)	Promotes chondrocyte survival, proliferation, and biosynthesis of cartilage matrix macromolecules; anti-inflammatory effects [88,89]	Targeting and improved residence time	[90]
Hollow mesoporous silica nanoparticles capped with chitosan	260.76–290.17 nm	Celastrol	Celastrol can block the secretion of IL-1β and TNF in OA animals, celastrol can eliminate the infiltration and proliferation of immune cells and prevent cartilage and bone damage [91,92]	pH-responsive, huge loading capacity, and good biocompatibility	[91]
Poly(2-methacryloyloxyethyl phosphorylcholine)-grafted mesoporous silica nanospheres (MSNs-NH_2_@PMPC)	180–260 nm	Diclofenac sodium	Analgesic and anti-inflammatory effects by inhibiting various enzymes and blocking the synthesis of prostaglandin. [77]	Super lubricating effect, high drug load, and slow release	[93]
Azobenzene-modified mesoporous silica nanoparticles with β-cyclodextrin-modified poly(2-methacryloy- loxyethyl phosphorylcholine) (bMSNs-AZO/DS/CD-PMPC)	Ca. 100 nm	Diclofenac sodium	Analgesic and anti-inflammatory effects by inhibiting various enzymes and blocking the synthesis of prostaglandin. [93]	Light-responsive drug release and super lubrication property	[94]
Gold nanoparticles	20 nm	Gold (Au) compounds	Suppression of lysosomal enzyme release by the phagocytic cells, modulation of prostaglandins, the suppression of synovial cells proliferation, and collagen synthesis [95,96,97,98]	Better therapeutic effect	[95]
PLA-Cy7	10–25 µm	KGN	Enhance cartilage regeneration of bone marrow mesenchymal stem cells (BMSCs) [99,100]	Cartilage protective effect and a high drug loading	[99]
MIL-100 (Fe)	Around 100 nm	Protocatechuic acid (PCA)	Anti-inflammatory and antibacterial, downregulate the indicators of inflammatory factors, including inducible nitric oxide synthase (iNOS), cyclo-oxygenase-2 (COX2), and metalloproteinase with thrombospondin motifs (ADAMTSs) [101]	High loading, good biocompatibility, and pH responsiveness	[101]
Liposomes					
HSPC and DOTAP	500–900 nm	6-methoxy-2-naphthylacetic acid (6-MNA) and its double salt with DSPE	A nonsteroidal anti-inflammatory drug, a potential and selective inhibitor of cyclo-oxygenase-2 enzyme [70]	Good biocompatibility, a long half-life (21–27 h), and tendency to penetrate well into synovial fluid	[70]
DSPC, cholesterol, and octadecylamine	135.17 ± 28.3 nm	Rapamycin	The potential therapeutic effect of rapamycin is the mammalian target of rapamycin (mTOR), which regulates many cellular processes, such as growth, proliferation, and protein synthesis to reduce the severity of osteoarthritis [102,103,104]	Together with low-intensity pulsed ultrasound (LIPUS), reduced drug dose and administration frequency	[102]
Gels					
Chitosan microspheres (CMS) in hydrogel of photo-crosslinked gelatin methacrylate (GelMA)	ca. 100 µm	Sinomeniumis	The level of matrix metallopeptidase 13 (MMP-13) protein, a marker of cartilage degradation in rats, was decreased through the NF-κB signaling pathway, the pathogenesis of collagen-induced arthritis was blocked, and the expression of MMP13 was down-regulated; regulating autophagy, and attenuating the release of inflammatory cytokines [105,106,107,108]	Low degradation rate and high swelling ratio of GelMA hydrogel, controlled release of drugs	[105]
Three-dimensional (3D) gelatin-based microcyrogel	-	Mesenchymal stem cells (MSCs)	Anti-inflammatory and pro-regenerative paracrine functions of the MSCs within the lesion site [109,110]	Minimized cell dose while retaining therapeutic effects	[110]
HA, poloxamer 407	-	HA, and diclofenac potassium (DK)	Joint lubrication, inhibiting various enzymes and blocking the synthesis of prostaglandin [77,111]	Extended release time of drug, thermo-responsive	[111]
PLGA MPs in acellular agarose hydrogel	46 ± 17 µm	Dexamethasone (DEX)	Anticatabolic and pro-anabolic effects on cartilage [112]	Targeted, low DEX dose carrier with improved effects	[112]
PEG-4MAL microgel, PLGA NPs, synoviocyte- or cartilage-targeting peptides	50.4–51.1 µm	Model small molecules	-	Long retention time (>3 weeks) in the joint, synoviocyte- or cartilage-targeting	[113]
Lectin-cholesterol liposome in cross-linked gelatin methacryloyl (GelMA) microgel	75–145 µm	KGN	Enhancing cartilage regeneration of bone marrow mesenchymal stem cells (BMSCs) [114]	Slow release of KGN	[114]
Alginate microspheres in thermosensitive chitosan and β-glycerophosphate hydrogel	10.744 ± 1.246 µm (alginate microparticles)	Diclofenac sodium	Inhibiting various enzymes and blocking the synthesis of prostaglandin [77,115]	Thermo-sensitive, sustained drug release (5 days), biocompatible, superior anti-inflammatory effect	[115]

PDI: polydispersity index.

## Data Availability

Not applicable.
